# Recycling Lithium-Ion Batteries—Technologies, Environmental, Human Health, and Economic Issues—Mini-Systematic Literature Review

**DOI:** 10.3390/membranes14120277

**Published:** 2024-12-21

**Authors:** Geani Teodor Man, Andreea Maria Iordache, Ramona Zgavarogea, Constantin Nechita

**Affiliations:** 1 Analytical Chemistry and Environmental Engineering Department, University Politehnica of Bucharest, 011061 Bucharest, Romania; geani.man@icsi.ro; 2 National Research and Development Institute for Cryogenics and Isotopic Technologies—ICSI Ramnicu Valcea, 4 Uzinei Street, 240050 Ramnicu Valcea, Romania; ramona.zgavarogea@icsi.ro; 3 National Research and Development Institute for Forestry “Marin Drăcea”—INCDS, 128 Boulvard Eroilor, 077190 Voluntari, Romania

**Keywords:** lithium toxicology, lithium-ion batteries recycling, electromigration process, membrane processes, electromigration process, circular economy

## Abstract

Global concerns about pollution reduction, associated with the continuous technological development of electronic equipment raises challenge for the future regarding lithium-ion batteries exploitation, use, and recovery through recycling of critical metals. Several human and environmental issues are reported, including related diseases caused by lithium waste. Lithium in Li-ion batteries can be recovered through various methods to prevent environmental contamination, and Li can be reused as a recyclable resource. Classical technologies for recovering lithium from batteries are associated with various environmental issues, so lithium recovery remains challenging. However, the emergence of membrane processes has opened new research directions in lithium recovery, offering hope for more efficient and environmentally friendly solutions. These processes can be integrated into current industrial recycling flows, having a high recovery potential and paving the way for a more sustainable future. A second method, biolexivation, is eco-friendly, but this point illustrates significant drawbacks when used on an industrial scale. We discussed toxicity induced by metals associated with Li to iron-oxidizing bacteria, which needs further study since it causes low recycling efficiency. One major environmental problem is the low efficiency of the recovery of Li from the water cycle, which affects global-scale safety. Still, electromembranes can offer promising solutions in the future, but there is needed to update regulations to actual needs for both producing and recycling LIB.

## 1. Lithium Resources and the Need for Recovery

The energy transition at the forefront of EU politics and energy markets is evolving, and green energy, including electric propulsion, is increasing interest. Electric vehicles, as well as electronic equipment and devices in continuous development, use the energy density of rechargeable batteries [1]. The lithium-ion battery is an existing energy storage solution. The global battery cell capacity market has continuously grown globally, recording almost 845 Gigawatt-hours (GWh) in 2020 with an estimated growth potential of 3 TWh in the next ten years [2]. Global lithium (Li) reserves are quantitatively significant, approximately 21 million tons, apart from the 86 million tons coming from resources. Even so, the 82,000 t of lithium from production reported in 2020 did not cover the lithium needs given by global market demand [3]. Li is known as a metallic element with the lowest relative atomic mass, a slightly alkaline metal, which is present, especially in the composition of batteries [4]. It has excellent chemical and physical properties, given its high specific heat capacity, good thermal conductivity, low electronegativity, and low electrical resistance. According to the United Geological Survey 2022, Li as a global resource was estimated at approximately 89 million tons [4]. In nature, lithium is not found in its pure metallic form, occurring in several representative forms in the form of deposits, as follows: clay (7%), brine (58%), zeolite (3%), hydrocarbon brine (3%), hydrothermal salts (3%), and pegmatites (26%) [5,6]. In 2020, the USGS report shows that the largest share of Li production was from brine, reaching 50% of the global output [7]. The representative Li deposits are pegmatites (26%), sedimentary rocks (8%), and brine associated with evaporite deposits (66%) [8].

The International Energy Agency estimates that Li demand will increase by almost 90% in the next 20 years [9]. Li and its isotopes have various applications: electronic products, ceramics, accelerator or cement additives, defense and aviation industries, polymer production, pharmaceutical industry, nuclear industry, aluminum production and continuous casting, lubricants, air treatment, and other fields, and in the case of batteries. Li compounds were found in optical equipment due to their corrosion resistance, high refractive index, nonlinear optical effects, and temperature resistance [10]. This highly reactive element is ubiquitous, from electric vehicles to portable devices to industrial equipment or materials [11]. Also, in the nuclear industry, Li is used to produce tritium for atomic fusion [12]. In the form of the Li⁷ isotope, it is used as a thermal agent to cool nuclear reactors and in other applications in this industry [13]. The literature illustrates a significant increase in Li in various industries, mainly in vehicle batteries, accentuating that metal reserves are insufficient for the next generation [10]. Thus, the correct recycling of waste resulting from exploitation is the focus of researchers in laboratory and industrial conditions, seeking the most efficient methods [14,15].

An overview of the literature screened illustrated a significant interest in LIBs’ components and pathways to Li recovery, which has become of great interest in developing innovative Li-based battery recycling technologies [16,17]. Even so, different limitations of actual methods require increasing interest in developing new techniques to recover metal elements [18]. Here, we will discuss the technology used for Li production, toxicity and the biomedical implications of Li, the effects of pollution with Li-ion batteries, and the most used methods for recycling. Also, we present a perspective on technical solutions for more efficient recovery, recycling, and removal, especially of LIBs, using techniques conceptually similar to “urban mining”. The study objectives were to synthesize the degree of knowledge regarding (i) the presence and recovery of lithium from stored waste and present in the environment, (ii) the degree of Li toxicity derived from Li waste, (iii) human health risk assessment associated with Li occurrence in environment and food, (iv) lithium speciation and aqueous solutions with Li, and (v) membrane processes with Li-component, and (vi) the economic aspects related to LIB recycling. We analyzed and identified ways of integration into classical technologies for isolation removal and recovery of lithium and perspectives for electro-membrane processes. Even if various studies evaluated the LIB recycling technologies, we herein produced a synthesis that almost equally combines the technologies and human/environmental issues caused by Li and LIB recycling. Also, we pointed out the recycling aspects related to a green economy, which must be interconnected to present specific regulations for citizens and countries. The truth regarding producing LIB and recycling is that new technologies can solve environmental issues.

## 2. Literature Interest in LIB Recycling

**Figure 1:**
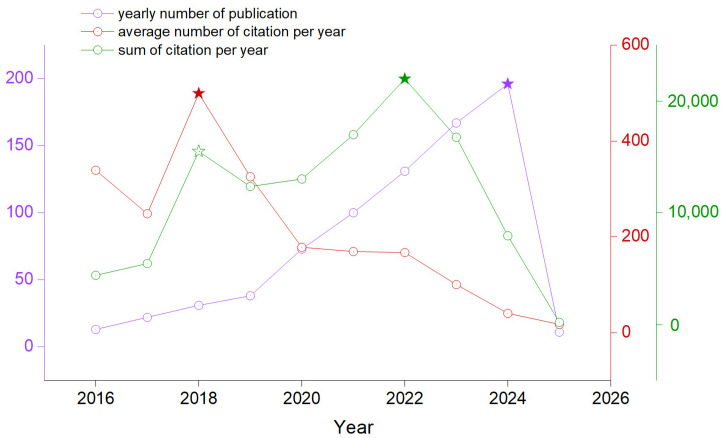
The number of articles published yearly is based on the imposed interrogation criteria in the WOS online database and the cumulative number of citations per year.

The Web of Science online database was interrogated using the following query: “*lithium-ion batteries recycling* and *lithium recovery* and *lithium removal* and *membrane lithium separation* and *membrane lithium recovery* and *membrane lithium removal* and *lithium recycling*” in the abstract. A database including 782 articles since 2016 was selected using as relevance a filter of at minimum ten citations for each article, except for those published in 2024 and 2025. Since 2016, when 13 publications corresponded to our criteria, the interest has grown faster, and the trend is still sustained. In 2024, the number of peer-reviewed articles published in the WOS online database arrived at 196, and those that occurred for the next year, 2025, had already been 11 (Figure 1). The number of citations had a decreasing trend, mainly due to the sparse goals of the research. When evaluating the article’s goal, which accounted for the highest interest of the audience each year, it was different. Since 2016, considerable interest has been invested in reviewing general concepts of processes and technologies for recycling and recovering spent lithium-ion batteries [19]. In deep mechanical separation, vacuum metallurgy and reductive ammonia leaching of metals from cathode scrap by sodium sulfite methodology of recycling metals from LIBs were also of great concern [20,21]. A key study discussed the 30 years of LIBs development and reasons for technological shifts, followed by a point of view regarding actual alternatives in current battery research [22]. Starting with 2019, the interest was focused on waste management battery recycling resulting from the end of life of electric vehicles (EV) [23,24,25].

The shifting of recycling technology from pyrometallurgy to hydrometallurgy to efficient direct and indirect recycling was a concept that gained attention [26,27]. Also, at this stage, the importance of recycling LIBs in a circular economy emphasized the positive aspects of reutilizing valuable metals [28]. The pretreatment methods for recycling active cathode metallic elements in pyrometallurgical processes were evaluated, and at the same time, there occurred interest in discussing legislation regarding this subject [29,30]. The maturity of recycling technologies can be achieved in 2022 since there occurred a new concept of evaluating environmental issues associated with different methods [31]. Future concepts will focus on green recycling discussed under needs imposed by the circular economy [32]. Under the current needs, predictions regarding future scenarios’ demand in lithium, cobalt, manganese, and nickel were evaluated to understand the importance of improving recycling potential [33]. Starting with 2024, a powerful interest was associated with improving solvents and membranes to increase performance and economic importance at a large-scale, environmentally friendly industrial level [34,35]. Approaches such as biomass pyrolysis gas reduction and bioleaching are promoted for future studies due to their capacity for energy-saving [36,37].

## 3. Lithium Toxicity for Environmental and Human Health

To the costs for green energy, lithium extraction, which uses sulfuric acid and sodium hydroxide, can be added, poisoning the soil and water and causing significant safety issues. The extraction of lithium from spodumene using the sulfuric acid method is the most common method in the lithium ore processing industry [38]. Another common way to extract lithium from brine uses membrane techniques, and the main methods are electrolysis, nanofiltration, membrane electrolysis, membrane extraction, liquid support membrane, ion-imprinted membrane, capacitive deionization, and membrane distillation crystallization [39,40]. The chemical reagents used and the amount of energy used increase production costs and pollution values [41]. Lithium-ion battery waste originating from residues or by-products is considered a narrow technological point, susceptible to separation and extraction operations [42].

**Figure 2:**
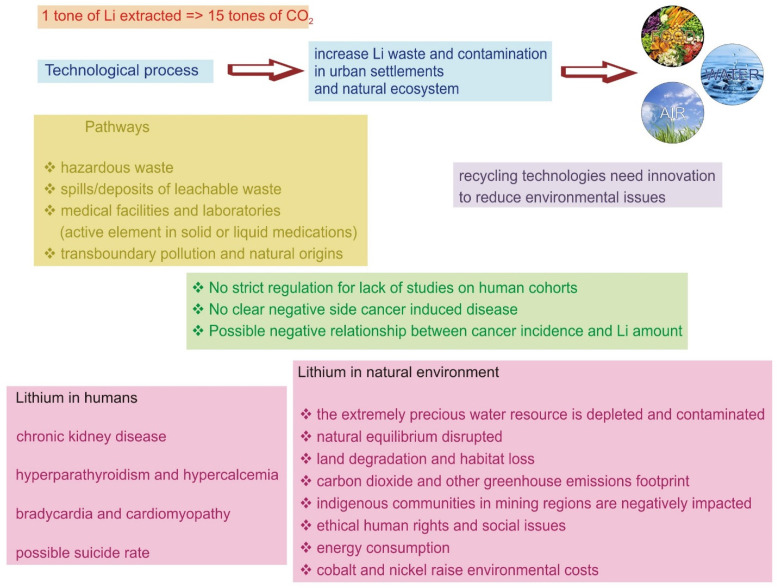
Schematic interaction between different Li sources of lithium and the effects of contamination in the environment and humans.

Also, the environmental issues are related to the fact that for the extraction of 1 tonne of mined Li, the emissions of CO_2_ are 15 tones. At this ratio, we must add the emission used mainly from the fossil energy used to process the raw materials until the final stage. Furthermore, if we consider the need to reach high temperatures for processing and China is the primary producer of LIB (around 70%), using coal as its primary energy source and the second natural gas, the footprint of CO_2_ is extremely high. Even more, the processes are conducted under high amounts of water, and the mines are located mainly in deficit regions with water supply. On the other hand, chemicals and waste resulting from exploitation drastically affect the surface and groundwater reserves. The Danish Environmental Protection Agency has classified lithium as hazardous, and the Australian Inventory of Chemical Substances has similarly listed the metal [12]. The highly volatile concentrations of this metal are present in a continuous increase in all ecosystems, including soil and water. A direct pathway was detected of lithium randomly occurring in various food products and drinking water, which are further ingested by humans, resulting in a potentially dangerous element from a global perspective [43,44]. A schematic representation of interactions and effects of the Li industry is presented in Figure 2.

Unfortunately, Li is not regulated through international laws or directives, so it is challenging to establish limits after which daily ingestion threatens life. Li can be assessed as an emerging environmental contaminant, part of the sustainable energy strategy, and is more frequently evaluated based on its contamination- and accumulation-associated risks [45]. Li is released into the environment and contaminates groundwater, water sources, and soil [46,47]. Possible poisoning processes in animals and humans are due to excessive lithium intake, which leads to various reactions, such as chronic kidney disease [48]. The two types of demonstrated side effects of Li on human health were found, first from long-term treatment and second from ingesting food and water with higher Li amounts. The first category of studies was found to associate long-term therapy with hyperparathyroidism and hypercalcemia [49], bradycardia and cardiomyopathy [50], and pulmonary toxicity and genotoxicity [51]. On the contrary, Li demonstrated a possible negative relationship between cancer incidence and its therapeutic effects on cancer [52]. When discussing large amounts of Li ingested via food or water, there were different associations between suicide rate and dementia [53].

Urban pollution with different equipment that contains Li in various structures (e.g., cameras, surveillance, alarm systems, personal mobility, solar energy storage, smart baggage, power tools, small electronic tools, and toys) is exposed to fire-related incidents [54]. However, industrial waste and more dangerous biomedical waste are complicated to evaluate, and further, there are new technologies for recycling. Lithium-rich elements that constitute waste have caused anthropogenic releases, which increase in volume, especially in urban areas, through aqueous lithium enrichment [45]. LiPF_6_, LiBF_4_, and LiClO_4_ from the cathode can cause the emission of toxic gases, pollute the air, and stimulate the body through inhalation and skin [55]. Li can be explosive and flammable in contact with air upon immediate and sudden exposure [56]. Thus, in the industry, workers can be exposed to accidental inhalation and/or long-time exposure to low amounts, which demonstrate side effects [57,58]. At a blood concentration of 15–20 mg L^−1^, lithium develops toxicity, leading to kidney problems, nausea, vision problems, or medical emergencies such as cardiac arrest or coma. Neurological, cognitive disorders, cardiovascular, gastrointestinal, cerebellar dysfunction, and, in some cases, persistent neurotoxicity were reported as a reaction to exposure to high Li amounts [57,59]. Considering the medical risks of assimilating excess lithium and its effects in exploitation or release into the environment above the permitted limits, precautionary measures must be taken in its recovery or isolation circuit [60]. The absence of a complete lithium recycling circuit leads to the elimination of various materials in the ecosystems, which, at the end of the chain, will affect at least the quality of human life quality [57]. Innovation and sustainable practices are needed to find sustainable advances in LIBs recycling to minimize row metal extraction.

## 4. Lithium-Ion Battery, Component Elements

An electrochemical energy storage device, a Li-ion battery incorporates several main elements: anode, cathode, electrolyte, separator plastic, binder material, and current collector [55,61]. Depending on the type of cathode, this type of battery is differentiated by the type of metal oxide (Li) used [29,62,63]. Recent studies qualify Li titanate (Li_2_TiO_3_) as a cathode material with promising properties [64]. Conventional batteries have an anode of graphite, which is present in the structure at a nanometer size [65]. The Li-ions reach the anode and are integrated into the micropores, and the amount of lithium ions thus assimilated has a direct relationship with the battery’s charging capacity [66]. The electrolytes between the opposite electrodes are the medium that supports the movement of lithium ions [67]. The most commonly used cathode materials are Li-manganese oxide, lithium cobalt oxide, lithium nickel oxide, and lithium iron phosphate, as well as (layered) cathode materials: lithium nickel cobalt aluminum oxide or lithium nickel cobalt manganese oxide [68,69]. The graphite in the anode is characterized by high electrical conductivity, cyclic stability, reversible theoretical capacity—up to 372 mA/hg, increased coulomb efficiency, and a good platform for charge–discharge processes [70]. In Li-ion batteries, the current collectors are metals (e.g., Cu, Al, for anode/cathode) with high electrical conductivity [71]. Microporous polymers are the most common solutions for Li-ion battery separators, with polypropylene and polyethylene being successfully used materials. Frequently used, liquid electrolytes contain (a) small molecules (lithium salts) and (b) organic solvents that dissolve lithium salts. Solid–liquid contact occurs through the interface of the material from which the battery electrode is constructed and the liquid electrolytes. Solid-state electrolytes can be inorganic (sulfides, oxides, halides), composites (inorganic fillers combined in an organic matrix), and polymer (based on Li salts that are dissolved in a polymer matrix) [72]. The solid electrolyte is resistant to high temperatures; is non-corrosive, non-volatile, non-explosive; and has low reactivity with lithium metal. Still, it can also stop the growth of lithium dendrites [73]. Inorganic Li conductor fillers are preferred, as they significantly increase the mechanical properties and transfer number [74].

## 5. Environmental and Human Issues Generated LIBs

The end-life of Li-ion batteries, in most situations, refers to the loss of Li content and the partial or total loss of active materials in the negative/positive electrodes and other active chemicals [75]. Human-induced toxicity generated by Li-ion battery waste released into the environment is associated with toxic substances released in the soil, water, and air [76]. The graphite anodes of used lithium-ion batteries (synthetic graphite or natural graphite), through incineration or storage as waste, lead to environmental pollution with dust, but also with a greenhouse effect [77]. The electrolyte (LiBF_6_/LiBF_4_) is an emergent pollutant resulting during combustion or heating processes, affecting human health as dust inhalation [78]. When heated, the electrolyte is corrosive, and the binder used in lithium-ion batteries (e.g., polyvinylidene fluoride/polytetrafluoroethylene) produces dangerous gas [79]. Being easily hydrolyzed, lithium salts produce gases hazardous to humans when in contact with water [66]. Classic Li-ion batteries use flammable organic liquid electrolytes, which can leak into the environment [80]. Leakage and volatilization of the electrolyte can lead to decomposition or hydrolysis, resulting in dangerous gases such as arsenic, fluorine, tiny molecules of organic matter, phosphorus compounds, methanol, ethanol, acetaldehyde, and formic acid [81]. Recent studies showed that cathodic oxide-LIB-(species: aluminum foil, cobalt oxide, nickel oxide, and manganese) can seriously affect the respiratory system and the skin [78]. The presence of toxic chemical compounds, in specific quantities, reaching groundwater or wastewater can cause serious health problems and irreversibly affect the environment [82]. The impact on the environment is also generated by the improper storage or disposal of lithium-ion batteries, which are potentially explosive due to the reactivity of the electrode components [83]. Binders and fluorine-containing electrolytes are significant organic pollutants in lithium-ion battery recycling [84]. In electronic waste management, including used batteries, exposure to metals or hazardous substances of a toxic nature poses a danger to humans due to stored contaminants, such as fatty acids. Pollution with waste and non-compliant recycling leads to contamination of various matrices such as groundwater/surface water, soil, and air [85]. Used lithium-ion batteries, accumulated in warehouses or stored randomly, devastate the environment through possible incineration processes (due to the various processing/recycling techniques used as raw material) and uncontrolled storage that favors their accelerated deterioration. Thus, industrial recycling and environmental protection processes must be necessary, mandatory, and strictly regulated [86].

## 6. The Main Methods of Recycling Li-Ion Batteries for Metal Extraction

Debates on battery recycling by various methods focus on the possibilities of net reduction of environmental impact due to the intensity of the techniques used (energy and chemical). The accumulated information on battery recycling is widely discussed in the literature, with various aspects being reported to government agencies to regulate battery manufacturing and recycling [87]. Lithium-ion batteries undergo three distinct phases in the recycling process: pretreatment, valuable metal extraction, and the end product [83]. Few pilot plants recover Li from waste batteries, the recovery rate being less than 1%, a percentage influenced by high operational costs, low amount of Li in waste, and high energy consumption [88]. Used batteries can be recycled directly or indirectly [34]. In direct recycling (without disassembly), a simple approach involves sorting the waste and performing physical treatment, recovering the materials, eliminating the residues (waste), and extracting recyclable materials and metals that have commercial value [89]. If indirect recycling is used, the batteries are dismantled for secondary use. Most of the time, the composition of LIBs is 5–20% Co, 5–7% Li, 7% plastic materials, and 15% organic and chemical substances [90]. Recycling processes through methods such as pyrometallurgy, hydrometallurgy, metallurgy, or direct recycling use different methods and techniques for lithium recovery, with specific costs, yields, and results, obtaining various products [91].

### 6.1. Hydrometallurgical Process—Flexible and Versatile for a Wide Range of Metal Ores and Recovery of Secondary Sources

During the hydrometallurgical stages, metals are refined and extracted in aqueous environments [92]. This process is primarily related to the solid–liquid ratio, temperatures, run-off periods, and unnecessary species, correlating with the isolation of metals with good purity and quality [93]. The hydrometallurgical recycling method includes leaching episodes (deep eutectic solvent, bioleaching, acid leaching) [65]. Hydrometallurgy uses low-temperature processing technologies (e.g., solvent extraction, leaching, electrowinning, and precipitation). The specific steps of the process are leaching the metal from the concentrate or ore (using alkali or acid), isolating the metal from the leaching solution by extraction or precipitation (using a solvent), and electrically isolating the metal present in the purified solution [94]. Chemical leaching or extraction using hydrometallurgy, having a high utilization rate, uses reagents in the process [nitric acid (HNO_3_), hydrochloric acid (HCI), hydrogen peroxide (H_2_O_2_)] to separate/extract metals from cathodes. The working temperature is generally below 100 °C, and Li can be recovered along with other transition metals. These hydrometallurgical processes efficiently obtain pure metal(s) salts from disused batteries, using reduced energy in the process, which also results in secondary products (salts) [95]. Being a versatile method in treating impurities, the method is environmentally friendly and relatively economical. This process is primarily used to recover valuable metals selectively, and compared to the second-use pyrometallurgical method (which consumes energy, involves high investment costs, and produces worthless residues), it is much more efficient [96].

Considering the share of costs in battery production, this is exceptionally high in the case of cathodes. This aspect is the starting point for more research to recover economically valuable metals from waste cathode active elements LiCoO_2_ by hydrometallurgical means. Standard techniques include acid leaching, crushing, solvent extraction, precipitation, and physical separation [97]. The expensive part of LIBs, consisting of cathode active material, determines that recycling is more advantageous than mining. Thus, the purity of the recovered cathode materials always creates perspectives and challenges in the technical recycling approach [98]. The cathode powder, which incorporates the most valuable metals, is obtained by crushing/disassembling [99]. Several hydrometallurgical processes are found in the literature, a significant part of which goes through stages of crushing whole cells, and another part has electrodes that have been separated, being present in leaching–precipitation processes and stages [100]. The hydrometallurgical process can select lithium in the last stage of recycling, and the lack of commercialization of this process on an industrial scale does not reflect the increased recovery potential through this technique [88].

### 6.2. Pyrometallurgical Process

Pyrometallurgical processes are used to reduce metal oxides to an alloy, and technological steps include using a high-temperature furnace [101]. High temperatures recover valuable metals, which are subsequently purified through various chemical/physical transformations, depending on factors such as processing time, temperature, flux addition, and types, as well as the purging of gas [29]. The available literature discusses various approaches to pyrometallurgical technologies that do not efficiently recover from used LIBs, except metals such as nickel, manganese, or cobalt [102]. Pyrometallurgical recycling by standard methods is inefficient in recovering Li due to costs. At a working temperature of 1000 °C, Li is lost through the generated slag and the combustion dust resulting from the process [88]. Previous reports have shown that chlorination roasting successfully extracts Li from the remaining slag, followed by a water-leaching process [103]. Even though pyrometallurgical processes simplify processing, yield is higher, and installation is simple (compared to the hydrometallurgical process), the method relies on carbon-based reductants. This dramatically increases the energy consumed and significantly raises the CO_2_ in the atmosphere [104]. Electric arc furnace (EAF) dust occurs when remelting LIB waste (in an electric arc furnace) [105]. Pyrometallurgical technologies using high temperatures (e.g., pyrolysis, melting, or roasting) aim to recover elements from the cathode material. Li and Mn are enriched in the resulting residue in LIBs, while Ni and Co are concentrated (in alloys) after the melting process [106]. On a global scale, industrial pyrometallurgy has been developed by various companies in the following ways: high-temperature melting recovery process (HTMR), ultra-high temperature melting technology (UHT), and high-temperature calcination process [107]. At a commercial scale, pyrometallurgical installations can recover valuable metals, and the technological processes usually used cannot recover Li, plastic, and electrolytes from LIBs [108].

### 6.3. Biometallurgical Process (Biolexivation)

Biometallurgical leaching consists of dissolving metallic components from end-life LIBs using inorganic/organic acids of metabolites (microbial) [109]. Biometallurgy is a process that combines biology, metallurgy, and chemistry, the efficiency of this process being given by the ability of microorganisms to obtain soluble forms through transformation processes of solid compounds (insoluble) [83,110]. The redox chemistry of dissolved metal(loids), as well as insoluble minerals, can be significantly influenced by microbes [111]. Lithium, nickel, cobalt, and manganese can be obtained by bioleaching the cathodes of LIBs, mainly using different bacteria such as iron-oxidizing bacteria (*Leptospirillum ferriphilum*), sulfur-oxidizing bacteria (*Acidithiobacillus thiooxidans*) [83]. Other bacteria studied in biometallurgical processes are summarized in Table 1. Anyway, many authors do not specify the reaction conditions that are different for each case in part; bioleaching processes have different results in lithium recovery. The bacteria required for biometallurgical processes are challenging to incubate, and the specific treatment period is often long [112]. Recovery through biometallurgy has easy reaction conditions for a low bioleaching yield (bacteria challenging to cultivate) and presents economic advantages [79]. Having a low energy consumption compared to pyrometallurgy or hydrometallurgy, the biometallurgical process presents mild conditions in the specific stages, being preferred for environmental safety reasons. However, the slow kinetics do not recommend it for various applications [113].

## 7. Membrane Processes for Li Recovery from Liquid Electrolytes and Leaching Solutions or Waters Contaminated with Li

### 7.1. Membrane Processes

Ultrafiltration, microfiltration, reverse osmosis, and nanofiltration are specific techniques for treating water for reuse, but they are also found in applications for objectives of industrial interest [122]. Although they have two significant disadvantages related to the high pressure for periodic cleaning and the impossibility of separating any organic pollutant (dissolved in water), ultrafiltration membranes and spatial processes have been extensively studied to remove a high yield of wastewater [123]. Microfiltration membranes can remove some virus species, suspended solids, and bacteria at a specific pore size between 0.1 and 10 µm. Being a particle separation process at an applied pressure of approximately 2 bars, it uses membranes that behave well at high temperatures, corrosion, and pressure [124,125]. The lowest technological filtration pressure and the large size of the pores characterize them. Microfiltration succeeds in the size of the pores, separating several types of macromolecules [126,127].

Nanofiltration achieves the separation effect with the help of the membrane using hydrostatic pressure. The nanofiltration membranes are characterized by a pore diameter of 0.5–2 nm, specific process pressure of 10–15 bar, and molecular weight ≈ of 400–500 Da; they retain neutral species (molecular weight 200–300 g/mol) under certain conditions, and inorganic ions can be rejected [128,129]. Due to the selective separation mechanism, Mg^2+^/Li^+^ separation by nanofiltration has a high potential for development. The trade-off between Li/Mg selectivity and Li recovery faces difficulties, and nanofiltration membranes in a single-pass process proved inefficient [130]. The theoretical experiments consider that at four passes, nanofiltration with commercial membranes can arrive at a Li/Mg selectivity of over 4500 and more than 95% li recovery [130]. Utilizing a physics-informed deep-learning model for multi-ion membrane transport showed high performance for solving such problems in the future [131]. Nanofiltration avoids using chemical reagents, works relatively simply, consumes little energy, causes separation at a good yield, is an ecological process, and is economically profitable [132,133,134]. The currently available reverse osmosis technology applied to drinking water and industrial processes has evolved due to the development of robust membranes and energy recovery processes [135]. Reverse osmosis is energy-consuming, and membrane dirt is removed by frequent chemical cleaning; the working pressure is 15–150 bar [136]. Selective separation, antifouling, and improving acid–alkaline resistance with industrial applications make the membrane usable in various configurations [137].

### 7.2. Electromembrane Processes, Main Categories in Obtaining Li

In the case of ocean waters, we find Li in the range of 0.1–0.2 ppm, which is a low level. No technique separates lithium from ocean waters, making it advantageous and commercially attractive. When the concentration of Li increases in the range of 10–20 ppm (naturally found in geothermal brines), extraction processes become profitable even if the presence of other metal ions increases the complexity of the process [138]. Various methods of extracting Li from saline lakes can be found in the literature. The most common methods are extraction, membranes, precipitation, and absorption [139]. Some authors proposed a selective assembly of capacitive deionization based on the membrane principle [140]. Here, it is suggested that Li recovery electrode materials should be layered, possess high capacity, and have fast Li^+^ diffusion. The yield of ion separation, as well as their isolation from dirty waters, were analyzed and compared according to the performance of the MOFs (zirconium) in the composite membranes (PVDF) using UiO-66/ZIF-8/PVDF membranes. The process was found to have promising recovery performances [141]. Some research focused on membranes/nanofibers, which record excellent separation results with absorption [142]. Li^+^ ions can be precipitated and concentrated using membrane electrolysis if added to the cathode section, H_3_PO_4_. Contamination of the catholyte with divalent Mg^2+^/Ca^2+^ ions that induce phosphate salts with low solubility can be avoided if an appropriate cationic membrane is used. Membranes for recovery of Li from contaminated waters with a high content of metal ions through applications that use 2D nanomaterials have demonstrated good performance for separating Li ions from divalent ions [143]. The future perspective treats the selective recovery of Li with the help of the membrane of 2D nanomaterials functional in various filtration applications, especially of wastewater.

The good permeability and selectivity of membranes with large carboxylic functional groups—UiO-66-(COONa)_2_—have a characteristic related to ion/selective performance in that it can be modulated by the action of specific K^+^ or Mg^2^^+^ ions. It is a filter-adaptive membrane used for Li recovery from brines and lends itself to high salinities (0.5 M mg^2^^+^/ Mg^+^ ions) [144]. Recent research studied the selective extraction, from liquid solutions, of metal ions using tetrabutylammonium bromide membranes incorporated in the polymer for separation [145]. This membrane selectively separated Fe from Co, Ni, and Li from the liquid solution containing Co, Ni, and Li. Composite membranes with a selective recovery of Li compared to other ions and a high ionic conductivity show stability [146]. An approach regarding the perspectives of membranes with subnanometric pores is made by [147]. It treats the selectivity of different metal ions, which bring essential elements of the molecular mechanisms that influence the energy barrier of ion transport in subnanometric pores to the fore.

Some research proposes a mathematical analysis model for assessing LiOH production in various solutions using the generation of OH^−^ ions in the bipolar membrane. Even here, the migration of lithium during the transit through the cation exchange membrane process converges to obtain LiOH [148]. At the same time, the introduction of bipolar membranes creates conditions for generating OH^−^ and H^+^ ions without reagents. Elements with good productivity are obtained in separate rooms [149]. The good properties of the membrane and the purity of the received product (LiOH) were highlighted, along with the energy consumption and current efficiency. Some results show that in a symmetric-type electromembrane process, there is also a kinetic limitation related to the dissociation-water process, which can manifest with the recombination reactions of the dissociation elements located in the bipolar boundary zone [150]. Also, using a bipolar membrane in water-splitting electrolysis is an electromembrane-ecological process with high yield, from which bases and acids can result from salt solutions [151]. A bi-layer membrane (an ion-exchange membrane) is analyzed where the cation and anion exchange layers are combined. The bipolar membrane associated with electrodialysis was also investigated to treat sodium sulfate waste containing lithium carbonate [152]. Sodium hydroxide and sulfuric acid were obtained from sodium sulfate waste through transformation. It should be noted that, under advantageous technological conditions, the dirty waters obtained through purification were considerably well-preprocessed. Also, the analysis of heterogeneous ion exchange membranes was investigated, and the recovery of lithium and boron and their separation was carried out by electrolysis with the bipolar membrane from aqueous solutions with good recovery results [153]. The electromembrane process removes the associated chemical dosing for Li hydroxide. LiOH synthesis was used for an electrolyzer that has bipolar membranes. Ionic impurities were removed, and lithium chloride was isolated entirely [154]. The simultaneous production of LiOH and the elimination of divalent cations led to the association of monovalent selective membranes with bipolar membranes [155]. This association process puts into question a threshold in the simultaneous transfer of the three monovalent cations.

The literature proposes a mathematical analysis model for the assessment of LiOH production in a variety of solutions, using the generation of OH^−^ ions in the bipolar membrane, but also the migration of lithium during the transit through the cation exchange membrane processes that converge to obtain LiOH [148]. At the same time, the introduction of bipolar membranes creates conditions for generating OH^−^ and H^+^ ions without reagents. Elements with good productivity are obtained in separate rooms [149]. The good properties of the membrane and the purity of the obtained product (LiOH) were highlighted, along with the energy consumption and current efficiency. The asymmetric electromembrane process also has a kinetic limitation related to the dissociation-water process, which can manifest with the recombination reactions of the dissociation elements located in the bipolar boundary zone [150]. Using a bipolar membrane in water-splitting electrolysis is an electromembrane-ecological process with high yield, from which bases and acids can result from salt solutions [151]. A bi-layer membrane (an ion-exchange membrane) is analyzed where the cation and anion exchange layers are combined. The bipolar membrane associated with electrodialysis was also investigated to treat sodium sulfate waste containing lithium carbonate [152]. Sodium hydroxide and sulfuric acid were obtained from sodium sulfate waste through transformation. For example, the conversion of sodium sulfate was almost 100% under an energy consumption of 1.4 kWh/kg, resulting in purities of 98.32 and 98.23% for sodium hydroxide products [152]. The analysis of heterogeneous ion exchange membranes was investigated, and the recovery of lithium and boron and their separation was carried out by electrolysis with the bipolar membrane from aqueous solutions with good recovery results [153]. The electromembrane process removes the associated chemical dosing for Li hydroxide. LiOH synthesis was used for an electrolyzer that has bipolar membranes. A recent study showed the efficiency of obtaining LiOH directly from brines with a high LiCl concentration under 0.77 current efficiency and an initial concentration of LiOH of 0.5 wt% and LiCl of 14 wt% [156]. Also, the ionic impurities were demonstrated to be removed, and lithium chloride was isolated entirely [154]. The simultaneous production of LiOH and the elimination of divalent cations led to the association of monovalent selective membranes with bipolar membranes [155]. This association process puts into question a threshold in the simultaneous transfer of the three monovalent cations.

Monovalent ion-selective membranes have high separation characteristics due to the loading properties and the specific structure [157]. Previous studies have shown that electrolysis technology for recycling Li-ion batteries discusses some problems specific to membranes with high selectivity to Li [158]. Using a selective monovalent membrane (of ions), the distinct characteristics related to the transport between Li and divalent cations are the reason that leads to the precipitation of divalent cations compared to the membrane porosity. This phenomenon is caused by the limiting current, which decreases significantly during electrolysis. However, this process lends itself to the Li recovery. The electromigration of ions, accompanied by its characteristics in a selective monovalent process, is frequently used in industry to obtain refined salts from brines or seawater and for the selective recovery of metals from various industrial processes [159]. The essential separation mechanisms for monovalent ion-selective membranes are dehydration/rehydration, exclusion size, and exclusion, which are Donnan effects [157]. The fractionation of Mg^2+^ and Li^+^ ions by electrodialysis with monovalent selective ion exchange membranes in binary synthetic brine resulted in a high ratio of Mg/Li. Achieving a target selectivity is possible by using a membrane that has the necessary properties for the targeted ions. On the membrane, the formation of a dense layer and an opposite charge through a series of active type layers leads to excellent selectivity properties, leading to the limitation of the transfer, i.e., multivalent cations in the membrane due to the high electrostatic repulsion, but also the tightness of the layer (upper) [160].

Methods to produce LiOH from Li-contained brine were also evaluated at a lab scale using electro-electrodialysis with bipolar membrane using a pretreatment process with five cation and four anion exchange membranes. The result shows a high purity of Li_2_CO_3_ of 98% obtained with a process cost of 2.59 USD/kg at a current density of 30 mA/cm^2^ and feed concentration of 0.18 M [161]. The electrodialysis process demonstrated good performance, reduced energy consumption, and low pollution levels, and there are opportunities to be feasible at an industrial scale. Even though the process is almost ready for implementation, the membranes present various limitations, such as hydrophilicity, pore size, and charge, which determine the perm selectivity between Li and Mg [162]. A study using 1250 measurements of four feed salinities, three pH levels, and five current densities demonstrated that a standard binary solution neglects Na^+^ and K^+^ transport and overestimates Li^+^/Mg^+^ selectivity up on 250% and underpredict energy consumption with a factor of 54.8 [163]. Almost in each case, an equilibrium must be found between counterion selectivity and thermodynamic efficiency, which can improve the process with 6.25 an increase in Li^+^/Mg^+^ selectivity [163]. The separation of Li^+^ from Mg^2+^ remains the critical phase of Li extraction, and at experimental design, cation exchange membranes with dense surfaces demonstrated superior performance for Li/Mg separation [164].

### 7.3. Electromigration Combined with Selective Electrodes with Lithium/Selective Crystallization

Mass displacement can be achieved by applying electric fields to materials, a phenomenon defined as electromigration [165]. Various electromembrane separation methods have been studied; the hybrid techniques prove their efficiency for separating binary mixtures of Li^+^/K^+^, Li^+^/Ca^2+^, and Li^+^/Na^+^ ions for Li isolation (extraction) [149]. During the electromigration process of counterflow cations, the less mobile cations possess lower electromigration speed characteristics, passing through the porous membranes faster than the more mobile cations [166]. In Li-ion batteries, cathode materials can introduce Li^+^ from aqueous solutions. Following separation with an anion exchange membrane, the two similar electrodes, which have different degrees of intercalation (Li^+^), can reach synchronized/simultaneous operation [167]. The transit of metal atoms through a conductive medium refers to the diffusion of solids supported by the electric current. This process is possible if the current density has a value that favors a drift in the direction of the electron flow [168]. In the literature, there are approaches based on solution-diffusion–electromigration models supported by the perspectives of irreversible thermodynamics. Thus, the rejection of ions is revealed as a property of the transmembrane flow using the membrane permeation of salt ions (dominant) [169]. The model’s parameterization following the determination of the permeates from the electrolyte mixture (containing two salts) was used to interpret complex solutions of several electrolytes. They have a consistency similar to brine (with reverse osmosis) and seawater without any additional parameter similarities to the experimental results of the complex-type mixture [170].

The separation of some types of ions present in mixed solutions is possible due to the permselectivity of specific ions [171]. Electromigration makes it possible to isolate lithium isotopes at a high yield. Enrichment with ^7^Li (solid phase) of single crystals and the separation of Li isotopes by electromigration/crystallization association leads to good results [172]. In an aqueous solution, electromigration leads to easy separation in several stages due to the high maneuverability and specific reactions of the electrodes in this aqueous solution [173]. In some applications, if an electric field is applied to the electrolyte through electromigration methods, the compounds can separate, in the absence/presence of the support, under the direct influence of the electroosmotic flow and the electrophoretic mobility [174]. Classic anion exchange membranes and cations in the electrolysis process led to two product streams that are concentrated simultaneously and two other desalinated feed streams. In distinct concentrate chambers, the anions and cations from food streams can re-arrange into substances [175].

## 8. Recycling of End-of-Life LIBs in a Circular Economy and Regulation Framework

Various approaches have been regarding the economic feasibility of implementing LIB recycling technologies at an industrial scale, pointing out multiple aspects presented in the following lines [176,177,178,179]. The increasing interest in recovery Li, Mg, and Ni added a benefit, even if small, as we stated above in the description for each technology. LIB waste management was previously evaluated from the economic point of view, considering direct use, cascade use, and recovery [180]. A study evaluated the economic savings: USD 590 stationary, USD 480 direct reuse, and USD 50 recycling per LIB pack [181]. But the facts are not so simple since, if we go into detail, we can discuss real-world problems such as waste LIB purchasing and storage costs [182,183,184]. Afterward, we can discuss the technological costs of developing and functionalizing a recycling method and the supplies needed, such as chemical solutions [185,186]. Above all, serious discussions exist regarding carbon emissions and energy consumption for Lib recycling [187]. Other costs are associated with wastewater treatment, recycling residue, transport, and management infrastructure [188]. If we consider only those mentioned, we must calculate the environmental cost associated with using energy sources that are often non-environmentally friendly. Water is a scarce resource, and often, the waste and sludge resulting from being used in diverse recycling technologies threaten the ecosystem. A recent review of advances in lithium recovery from water resources illustrates that multiple studies present the results but only a few technical details [189]. This aspect is essential since it is challenging to synthesize the performance of each method and the diebacks versus advantages for the same and between methods. Thus, many studies search for improving processes such as re-use inspired by a circular economy [190] even if, in practice, it is complicated due to the accumulation of ions and floatation reagents [191,192]. Nowadays, new technologies closer to the environment, such as leaching agents, ultrasound, and microwave irradiation, can produce a high percentage of metal recovery [193]. Electrochemical ion pumping is another option that does not use chemicals for the regeneration of materials, reduces the consumption of water, and demonstrates a high capacity for recycling [194].

The circular economy sustained waste management after a hierarchy, and the waste resulted from end-of-life LIBs. A study conducted in the United States showed efficiency for both energy and valuable metals [180]. Another benefit is reducing eco-toxicology associated with landfill disposal bands for batteries if they are in cascade reused and recycled [94,180]. Based on recent achievements, it was stated that environmental impact can be attributed mainly to production and use stages up to 15%. A percentage of up to 80% is attributed to the whole chain, which explains the need for profitable industrial recycling methods [195]. In the coming years, we expect substantial waste, significantly threatening underdeveloped countries [196,197,198]. This knowledge improved the regulatory standards to cover consumers in the chain market, which has the obligation for the correct management of LIBS. This fact can be discussed only in industrialized countries [181]. At a global scale, the annual reports of LIB recycling market size were USD 8.10 billion in 2023 and 10.26 billion in 2024, and according to *The Economist* journal, recycling old cars LIB can cover the needs for new ones. The European Commission implemented a new LIB regulation in 2020, which includes footprint declaration and minimum information regarding recycling [199].

## 9. Final Remarks

Here, we evaluated the up-to-date knowledge regarding recycling lithium-ion batteries and recovering Li from aqueous electrolytes and the possible implications of health risks associated with Li contamination. Thus, various processes were discussed, and each method was documented mainly with limitations induced by each method. The biometallurgical process is an ecological process with a high environmental component, and from this perspective, it is advantageous compared to pyrometallurgy or hydrometallurgy. It has a low energy requirement and an advantageous process temperature. Even so, due to slow kinetics problems regarding the purity and quality of microbial cultures, microbes are also sensitive to the waste lithium-ion batteries for their toxic components. Complex waste flows influence microbial processes, which are reported in the literature as secondary effects of the processes. Even if microbial cultures were identified to have high lithium recovery at relatively long incubation periods, an insignificant volume of physical recycling was achieved on an industrial scale, posing an economic limitation that needs to be solved. Pollution with lithium-ion batteries is constantly increasing, and the existing recycling loops do not cover the need for recycling because there is a problem of balancing technological progress to achieve an environmental achievement. Aqueous electrolytes are a method that can potentially develop in the future. Future research directions must be designed for multi-cycle membrane equipment, which can differentiate ions and induce a scalable transport model, even if isolating other monovalent metal ions and Li opens new research directions. Even if ions have preferential mobilities, classic anion exchange membranes do not possess selectivity for ions with the same charge.

## Figures and Tables

**Table 1:** Examples of percentage of metal recovery using biometallurgical leaching.

Bacteria	Percentage of Metal Recovery (%)					Reference
	Mn	Cu	Ni	Co	Li	
*A. ferrooxidans*	20	N/A	N/A	*88*	100	[114]
	N/A	N/A	N/A	10	65	[115]
*A. ferrooxidans* *and* *A. thiooxidans*	N/A	N/A	89.4	50.4	99.2	[116]
*A. ferrooxidans*	N/A	N/A	N/A	94	60.3	[117]
*A. thiooxidans*	20	N/A	N/A	60	99	[118]
*Alicyclobacillus* sp. and *Sulfobacillus* sp.	N/A	N/A	N/A	72	89	[119]
*Aspergillus niger*	N/A	N/A	N/A	N/A	95	[120]
*Aspergillus niger* PTCC 5210	77	100	N/A	N/A	100	[121]

N/A not available data.

## Data Availability

All data are presented in the results section.
